# Australian bat lyssavirus infection in a captive juvenile black flying fox.

**DOI:** 10.3201/eid0503.990316

**Published:** 1999

**Authors:** H Field, B McCall, J Barrett

**Affiliations:** Queensland Department of Primary Industries, Moorooka, Australia.

## Abstract

The newly emerging Australian bat lyssavirus causes rabieslike disease in bats and humans. A captive juvenile black flying fox exhibited progressive neurologic signs, including sudden aggression, vocalization, dysphagia, and paresis over 9 days and then died. At necropsy, lyssavirus infection was diagnosed by fluorescent antibody test, immunoperoxidase staining, polymerase chain reaction, and virus isolation. Eight human contacts received postexposure vaccination.


### Australian Bat Lyssavirus Infection in a Captive Juvenile Black Flying Fox

**Hume Field,* Brad McCall,† Janine Barrett‡**
*Queensland Department of Primary Industries, Moorooka, Australia;
†Brisbane Southside Public Health Unit, Upper Mount Gravatt, Australia;
and ‡The University of Queensland, Brisbane, Australia

The newly emerging Australian bat lyssavirus causes rabieslike disease in bats and humans. A captive juvenile black flying fox exhibited progressive neurologic signs, including sudden aggression, vocalization, dysphagia, and paresis over 9 days and then died. At necropsy, lyssavirus infection was diagnosed by fluorescent antibody test, immunoperoxidase staining, polymerase chain reaction, and virus isolation. Eight human contacts received postexposure vaccination.

Australia was considered free of rabies and the rabieslike viruses of the genus *Lyssavirus* until the recognition in 1996 of Australian bat lyssavirus (ABL) as the cause of a rabieslike disease in a black flying fox *(Pteropus alecto)*(1) and a wildlife caretaker (2). While serotypic, antigenic, and sequence analysis show that ABL is closely related to classic rabies virus and European bat lyssavirus (1), phylogenetic analysis has clearly demonstrated that ABL represents a new genotype, genotype 7 (3). Australia is still considered free of terrestrial (genotype 1) classic rabies (4). Rabies vaccine and antirabies immunoglobulin protect laboratory animals against ABL infection (5), and their use pre- and post-ABL exposure is recommended for humans (6,7).

On the morning of December 8, 1997, two juvenile black flying foxes were found clinging to each other and vocalizing in a residential area near an urban flying fox colony. An experienced, rabies-vaccinated wildlife caretaker retrieved the two animals from an unusually low tree roost. On the basis of body weight and forearm measurements, their age was estimated at 2 to 3 weeks, an age of total maternal dependency. Their physical condition was normal.

Both animals, Bat 1 (male) and Bat 2 (female), remained with the original caretaker for 2 days before being placed with two different caretakers for hand-rearing. Bat 1 was communally housed with another orphaned black flying fox, and Bat 2 was housed with two others. For the next 5 weeks, all the bats were clinically normal. However, in week 6, Bat 1 began to exhibit signs of neurologic disease. The caretaker first observed the bat's sudden and progressive aggression toward its companion and separated them. Throughout day 1 of illness, the bat periodically "frothed at the mouth" and had repeated lordotic spasms, during which it vocalized loudly. Treatment with oral amoxycillin was initiated. On day 2, the bat was calmer but still vocal, attempting to bite objects and eating little. On day 3, it was no longer aggressive and was only able to eat pulped food and milk. On day 4, it was seen by a veterinarian, who noted severe pharyngitis, and administered injectable dexamethasone. The bat was much more alert that evening and ate solid food well. The dexamethasone injection was repeated on day 5; the bat remained alert and ate solid food overnight. On day 6, it was dysphagic and was again offered pulped foods and liquids. On days 7 and 8, it was unable to roost normally, lay supine, was progressively dysphagic, had diarrhea, and was losing weight. On day 9, it rapidly got worse and died. The carcass was submitted to the Queensland Department of Primary Industries Animal Research Institute for necropsy.

The emaciated carcass had poor pectoral muscle development and no perirenal, pericardial, or mesenteric fat reserves. ABL infection was diagnosed by fluorescein-labeled antirabies

Address for correspondence: Janine Barrett, School of Veterinary Science and Animal Production, The University of Queensland, Brisbane, Queensland, Australia 4072; fax: 61-7-3362-9420; e-mail: j.barrett@mailbox.uq.edu.au.

monoclonal globulin (CENTOCOR) in a fluorescence antibody test (FAT) on impression smears of fresh brain. With the absence of other lyssaviruses in Australia (1), a positive reaction with this lyssavirus genus-specific antibody is considered diagnostic for ABL. Sections of brain showed nonspecific, nonsuppurative meningoencephalitis with perivascular cuffs of mononuclear cells and widespread focal gliosis. Numerous neurons contained eosinophilic inclusion bodies, which are highly suggestive of lyssavirus infection.

Immunoperoxidase staining of formalin-fixed, paraffin-embedded sections of brain with a monoclonal antinucleoprotein antibody (Clone HAM, provided by R. Zanoni, Berne University, Switzerland) detected lyssavirus antigen in neurons of the frontal cortex, hippocampus, brain stem, and cerebellum, including Purkinje cells. This antigen distribution is consistent with previous reports of rabies (8). The diagnosis was independently confirmed at the Commonwealth Scientific and Industrial Research Organisation's Australian Animal Health Laboratory by FAT, immunoperoxidase staining, virus isolation in murine neuroblastoma cells, and sequence analysis of polymerase chain reaction (PCR) product (P. Daniels, pers. comm.). No blood was available for serologic testing.

After ABL infection was diagnosed in Bat 1, the Brisbane Southside Public Health Unit received information that up to eight persons had been bitten or scratched by the bat in the weeks before and during its illness. Six were bat handlers who had received postexposure treatment with five doses of rabies human diploid cell vaccine (HDCV) during a 1996 campaign that followed the diagnosis of the first human case of ABL (2,7). Two were unvaccinated members of the principal bat caretaker's household. Despite recommendations that unvaccinated members of bat caretakers' households not handle bats, the two had come into regular contact with the bat and may have been scratched during that time.

Lyssavirus prophylaxis was commenced in accordance with Australian recommendations (6,7). All eight persons provided blood for rabies serologic testing (indirect-enzyme linked immunosorbent assay [ELISA]). The six vaccinated bat handlers had titers of 1.130 IU/ml to >8.80 IU/ml 12 months after their initial vaccinations (World Health Organization–recommended protective level for rabies = 0.5 IU/ml [9]). Each of these received two further intramuscular doses of 1.0 ml of HDCV, according to the Australian recommendations for postexposure treatment of vaccinated persons. The two unvaccinated persons had titers of <0.13 IU/ml (nonimmune) and received the standard postexposure treatment for unvaccinated persons: 20 IU per kg of human rabies immune globulin (HRIG) by intramuscular injection and five intramuscular doses of 1.0 ml of HDCV. All eight remain well 10 months after the incident.

Throughout this episode, Bat 2 and the other three bats that had been directly or indirectly in contact with the infected bat remained healthy. After the diagnosis of ABL in Bat 1, these four animals were quarantined for observation at the Animal Research Institute for 11 weeks and then euthanized. All were antibody-negative (<0.5 IU/ml) by rabies rapid fluorescent focus inhibition test when quarantined and remained so during the observation period. Brain impression smears from the four were negative for lyssavirus antigen by FAT.

This case of naturally occurring ABL infection is of particular interest for several reasons. First, the astute observations of the bat caretaker provide possibly the first record of the clinical course of natural ABL infection in a flying fox. Second, to our knowledge, this is the first recorded case of ABL disease in a maternally dependent juvenile. Third, the case history provides the first indication of the incubation period and length of clinical disease in naturally infected flying foxes. Natural in utero infection with lyssaviruses is not known to occur (10), and the four flying foxes in contact with Bat 1 were unlikely to be the source of infection as they subsequently tested negative for ABL antibody and antigen. The infection appears to have occurred in the 2 to 3 weeks before the rescue and, after an incubation period of 6 to 9 weeks, produced 9 days of clinical disease. Infection in Bat 1 most probably resulted from a bite from an ABL-infected bat. This bat may have been Bat 1's dam.

This episode demonstrates the necessity of examining for ABL any flying fox that has bitten or scratched a person and of improving community and professional awareness of the disease and associated risks. Costly postexposure treatment with HRIG can be avoided if only vaccinated persons handle Australian bats.

### Acknowledgments

We thank Helen Luckhoff, Jill Nelson, Jennifer Hawyes, and Merle Thomas for reporting the case and making their records available; Barry Rodwell for performing the fluorescence antibody test; Natasha Smith and Craig Smith for technical assistance; Greg Smith, Ina Serafin, and Alan Westacott for performing ELISA (human) serology; Ross Lunt, Peter Hooper, and Alan Gould for fluorescence antibody test and immunoperoxidase staining, rapid fluorescent focus inhibition test (flying fox) serology, virus isolation, and sequence analysis of PCR product; and the general practitioners and the staff of Queen Elizabeth II Hospital for collecting serum samples and administering postexposure treatments.

Dr. Field is a veterinary research officer with the Animal Research Institute, Queensland Department of Primary Industries. His particular research interest is the epidemiology of wildlife diseases and the role of wild species as reservoirs of infection for domestic animals and humans. He is investigating the natural history of Australian bat lyssavirus and equine morbillivirus (Hendra virus), two recently emerged zoonotic diseases in Australia.

### References

1. Fraser GC, Hooper PT, Lunt RA, Gould AR, Gleeson LJ, Hyatt AD, et al. Encephalitis caused by a lyssavirus in fruit bats in Australia. Emerg Infect Dis 1996;2:327-31.
2. Allworth A, Murray K, Morgan J. A human case of encephalitis due to a lyssavirus recently identified in fruit bats. Commun Dis Intell 1996;20:504.
3. Gould AR, Hyatt AD, Lunt R, Kattenbelt JA, Hengstberger S, Blacksell SD. Characterisation of a novel lyssavirus isolated from Pteropid bats in Australia. Virus Res 1998;54:165-87.
4. Office International des Epizooties, 10 Nov 1998, [computer program]. Handistatus (download). Version 1.39. Microsoft Internet Explorer 4.0. http://www.oie.int/A_html.htm.
5. Hooper PT, Lunt RA, Gould AR, Samaratunga H, Hyatt AD, Gleeson LJ, et al. A new lyssavirus—the first endemic rabies-related virus recognised in Australia. Bulletin Institut Pasteur 1997;95:209-18.
6. Rabies and bat lyssavirus infection. In: Watson C, editor. The Australian Immunisation Handbook. 6th ed. Canberra: Australian Government Publishing Service; 1997. p. 162-8.
7. Prevention of human lyssavirus infection. Commun Dis Intell 1996;20:505-7.
8. Feiden W, Kaiser E, Gerhard L, Dahme E, Gylstorff B, Wandeler A, et al. Immunohistochemical staining of rabies virus antigen with monoclonal and polyclonal antibodies in paraffin tissue sections. Zentralbl Veterinarmed [B] 1988;35:247-55.
9. World Health Organization recommendations on rabies post-exposure treatment and the correct technique of intradermal immunization against rabies. Geneva: The Organization; 1997.
10. Constantine DG. Absence of prenatal infection of bats with rabies virus. J Wildl Dis 1986;22:249-50.

